# Incidence and clinical vital parameters in primary ketosis of Murrah buffaloes

**DOI:** 10.14202/vetworld.2015.1083-1087

**Published:** 2015-09-19

**Authors:** Ankit Kumar, Neelesh Sindhu, Parmod Kumar, Tarun Kumar, Gaurav Charaya, Surbhi, V. K. Jain, Sridhar

**Affiliations:** 1 Department of Veterinary Medicine, Lala Lajpat Rai University of Veterinary & Animal Sciences, Hisar - 125 004, Haryana, India; 2 Teaching Veterinary Clinical Complex, Lala Lajpat Rai University of Veterinary & Animal Sciences, Hisar - 125 004, Haryana, India; 3 Department of Veterinary Physiology and Biochemistry, Lala Lajpat Rai University of Veterinary & Animal Sciences, Hisar - 125 004, Haryana, India

**Keywords:** buffaloes, incidence, Keto-Diastix strip test, primary ketosis, Rothera’s test

## Abstract

**Aim:**

The present study was undertaken to ascertain the incidence and clinical vital parameters in cases of primary ketosis in Murrah buffaloes brought to teaching veterinary clinical complex, Lala Lajpat Rai University of Veterinary and Animal Sciences, Hisar and from adjoining villages of the district Hisar, Haryana, India.

**Materials and Methods:**

The investigation was conducted on 24 clinical cases (out of total 145 screened) of primary ketosis. The diagnosis was confirmed on the basis of clinical signs and significantly positive two tests for ketone bodies in urine (Rothera’s and Keto-Diastix strip test). Data collected were statistically analyzed using independent Student’s *t*-test.

**Results:**

Overall incidence of disease in these areas was found to be 16.55% and all the animals were recently parturited (mean: 1.42±0.14 month), on an average in their third lactation (mean: 2.38±0.30) and exhibited clinical signs such as selective anorexia (refusal to feed on concentrate diet), drastic reduction in milk yield (mean: 64.4±5.35%), ketotic odor from urine, breath, and milk and rapid loss of body condition. All the clinical vital parameters in ketotic buffaloes (body temperature, heart rate, respiration rate, and rumen movements) were within normal range.

**Conclusion:**

Primary ketosis in Murrah buffaloes was the most common seen in the third lactation, within the first 2 months after parturition with characteristics clinical signs and no variability in vital parameters. The disease has severe effect on the production status of affected animal.

## Introduction

Production diseases are mainly manmade problems, which occupy the most important place among the diseases of dairy animals as it directly or indirectly affect the economy of dairy farm and ultimately dairy farmers suffers from huge financial losses due to drastic decrease in production. Dairy cows and buffaloes are especially prone to production diseases in the last 3 weeks before parturition to 3 weeks after parturition which is also called as a transition period.

Ketosis is one of the most important production disorders caused by impaired carbohydrate metabolism leading to negative energy balance and consequently the excessive production of ketone bodies [1]. The disease is clinically characterized by sudden loss of milk production, selective anorexia, hypoglycemia, ketonemia, ketonuria, and low levels of hepatic glycogen [2,3]. Successful adaptation to milk production occurs due to coordinated changes in the body to support the dominant physiologic state of lactation. This orchestrated flow to a new equilibrium is defined as homeorhesis [4]. Negative effects (such as displaced abomasum, increased culling risk, lower milk production, and impaired reproductive performance) can carry into lactation [5-9], if animals are unable to adapt due to management factors, concurrent disease, or a multitude of other known and unknown reasons.

There are various risk factors for ketosis which may covers previous disease condition, breed, lactation, body condition score [10-12], season of calving, and dry period length [13-15]. Shortened dry period (35 days or less) reduces the risk for ketosis with little to no effect on production or reproduction in the subsequent lactation [13-15]. The disease chances increase from a low prevalence at the first calving to a peak at the fourth and in between farms, the cumulative lactational incidence varies greatly, averages about 40%, and can be as high as 80% in some herds [16].

The incidence of clinical ketosis has increased sharply in recent past due to steep increase in milk production of dairy animals. The majority of high producing dairy animals go through borderline ketosis during early lactation, and the most cases of ketosis occur within approximately 60 days of calving [17]. The present study was planned to study the incidence and vital parameters in clinical cases of primary ketosis.

## Materials and Methods

### Ethical approval

Ethical approval is not necessary for this type of study. However, animals were examined as per standard examination procedure, and samples were collected as per standard collection method without harming or giving stress to any animal.

### Place of study

The study was conducted in Department of Veterinary Medicine, College of Veterinary Sciences, Lala Lajpat Rai University of Veterinary and Animal Sciences (LUVAS), Hisar.

### Sample collection and clinical examination

The study was conducted on 24 clinical cases (selected out of 145 screened) of primary ketosis in buffaloes which were reported at Teaching Veterinary Clinical Complex (TVCC), LUVAS, Hisar and from adjoining villages of the Hisar district. A complete history of diseased animals with regard to age, sex, lactation chronology, stage of lactation, and milk yield was obtained from the animal owners or handlers. Eight apparently healthy buffaloes were also included in this study as a control group for comparison with primary ketosis affected animals.

### Clinical observations

Complete clinical examination of the suspected animals was made which included rectal temperature, pulse rate, respiration rate, and ruminal movements.

### Confirmatory diagnosis

**Figure-1:**
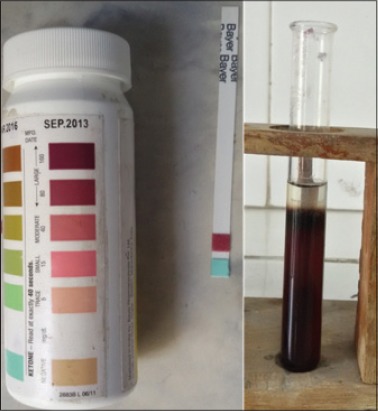
Positive result of Rothera’s test and Keto-Diastix strip test.

The diagnosis of primary ketosis was confirmed with the help of one qualitative (Rothera’s test) and one quantitative test (Keto-Diastix strip test) in urine. Purple color ring in Rothera’s test and the dark purple strip color in Keto-Diastix strip test indicate positivity of sample for primary ketosis (Figure-1).

### Statistical analysis

Data were expressed as mean (±standard error of the mean) and analyzed by applying independent Student’s *t*-test using SPSS statistical software.

## Results

The ailing buffaloes presented for treatment at the TVCC were screened for the incidence of ketosis. Of the 145 cases examined, an overall incidence of 16.55% (24 cases) was recorded for primary ketosis in Hisar and adjoining area during the study period. Animals having absence of any other concurrent diseases, selective anorexia (refusal to take concentrates) and a positive at least +++ test for ketone bodies in urine (Rothera’s test and Keto-Diastix strip test) were considered as a case of primary ketosis for this study. The age-wise incidence was recorded to be highest in the age group of 3-5 years (54.17%) followed by 6-9 years (45.83%) as shown in Table-1. Overall, it was found that most of the cases of primary ketosis occur in the age of 6 years (mean 5.71±0.33) as shown in Table-2. Similarly, the highest incidence of disease was seen in buffaloes in their first-third lactation (79.17%) followed by fourth-sixth lactation (16.67%) while only one animal was pregnant (4.16%) as shown in Table-1. Among the lactation stage, maximum number of cases were recorded during 0-2^nd^ month post-partum (91.68%) followed by 3^rd^-5^th^ month post-partum (4.16%) while only one case occurred pre-partum (4.16%) as shown in Table-1. So, overall it was also concluded that primary ketosis in buffaloes was most common encountered in their third lactation (mean: 2.38±0.30), within the first 2 months (mean: 1.42±0.14 month) after parturition as shown in Table-2.

**Table-1:** Incidence of primary ketosis in buffaloes with regard to age, lactation number and lactation stage (n=24).

Parameters	Age-wise (years)		Lactation no.			Lactation stage (month)		
		
	3-5	6-9	Pregnant	1^st^-3^rd^	4^th^-6^th^	Pre-partum	0-2	3-4
Number of cases	13	11	1	19	23	1	22	1
Percentage	54.17	45.83	4.16	79.17	16.67	4.16	91.68	4.16

**Table-2:** History (related to lactation) in buffaloes suffering from primary ketosis.

Parameters	Age (years)	Lactation chronology (No)	Lactation stage (month)	Milk yield (L/day)		Decrease in milk yield (%)

				Before illness	After illness	
Mean±SE	5.71±0.33	2.38±0.30	1.42±0.14	14.42±1.09	5.24±0.82	64.48±5.35

SE=Standard error

The first characteristic clinical sign observed in all clinical cases of primary ketosis was selective anorexia (refusal to feed on concentrates but continuance with other feed such as hay) and there was drastic reduction in milk yield (mean: 64.4±5.35%) as shown in Table-2. The anorexia varied from partial to complete depending on the severity and stage of lactation. The animals first refused to eat concentrates then ensilage and at last hay in severe cases with typical weight loss following decrease in appetite.

**Figure-2:**
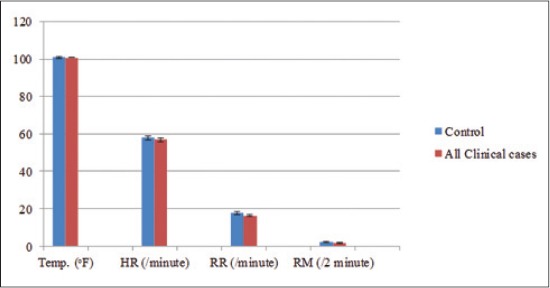
Graphical representation of a comparison between mean values of clinical vital parameters in healthy (n=8) and ketotic (n=24) buffaloes (Temp.=Temperature, HR=Heart rate, RR=Respiration rate, RM=Ruminal movements).

The body temperature (100.83±0.15°F), heart rate (56.88±0.87/min), respiration rate (16.29±0.41/min), and ruminal movements (1.62±0.19/2 min) were within the normal range and there was no significant difference when compared to control animals as shown in Table-3 and Figure-2. On rectal examination, feces were found to be scanty, mucoid, and dry in most cases. The buffaloes appeared dull and depressed with a sweetish ketotic odor in breath and urine in all the cases.

**Table-3:** Comparison between mean values of clinical vital parameters in healthy and ketotic buffaloes.

Parameters	Temperature (°F)	Heart rate (/min)	Respiration rate (/min)	Ruminal movements (/2 min)
Diseased animals (n=24)	100.83±0.15	56.88±0.87	16.29±0.41	1.62±0.19
Control animals (n=8)	100.94±0.32	57.88±1.02	17.62±0.62	2.00±0.26

*Significant (p<0.05), **Significant (p<0.01)

## Discussion

The results of the present investigation related to incidence of the disease on the basis of age, lactation chronology, and stage of lactation indicated that most of the animals were in their peak lactation phase. The age group of animals affected ranged between 3 and 9 years (mean 5.71±0.33 years). This indicated that animals affected were around 5-6 years of age, which is co-incidentally the peak lactation phase. The stage of lactation in ketotic animals ranged between 15 days and 3.5 months (mean: 1.42±0.14 month) which is also around the peak lactation stage. Similarly, chronologically the diseased animals were in their first to the sixth lactation. Reports from other authors [18-20] also support the outcome of the present study. Singh and Kasaralikar [18] recorded maximum number of clinical cases of ketosis in buffaloes of 8-9 years of age (57%) followed by 6-7 years (28%) and 10 years and above (14%) whereas Rautmare *et al*. [19] and Anantwar and Singh [20] observed the highest prevalence of clinical ketosis in buffaloes in fourth lactation.

Immediate periparturient period is of greater risk for ketosis which was also in accordance with the present study findings [2,21-27]. After parturition concentrate intake reaches to maximum 8-10 weeks after calving but peak milk production is at 4-6 weeks. This period of 4 (5-8) weeks leads to negative energy balance and generation of ketone bodies by mobilizing adipose tissue. In the current study, only one clinical case of ketosis was observed in a pregnant animal. Few authors [2,28] too reported that clinical ketosis is rare in late pregnancy. Most of the animals in present study were high yielders and their milk yield ranged between 6 and 20 L (average 14.42 L) before occurrence of disease and there was drastic reduction in milk yield following illness (mean decrease to be 64.48±5.35%). Clinically, ketotic buffaloes (n=24) exhibited clinical signs such as selective anorexia (refusal to feed on concentrate diet), drastic reduction in milk yield, ketotic odor from urine, breath, and milk and rapid loss of body condition, similar observation have made by several authors [2,18-20,29-37].

Various clinical vital parameters recorded in the present study such as temperature, heart rate, respiration rate, and ruminal movements were within normal range when compared to healthy control animals which was in agreement with earlier reports [35,36].

## Conclusion

Cases of primary ketosis can be confirmed by simple Rothera’s and Keto-Diastix strip test even at field level. The overall incidence of 16.55% was recorded for primary ketosis in Hisar and adjoining area during the study period. The disease was found to occur in the age group of 3-9 years (average approximately 6 years) which is generally the peak lactation phase. The animals were in their first-sixth lactation while only one animal was in advance pregnancy. The stage of lactation in ketotic animals ranged between 15 days and 3.5 months (average approximately 1.5 months). Ketotic buffaloes exhibited clinical signs such as selective anorexia (refusal to feed on concentrates diet), drastic reduction in milk yield, ketotic odor from urine, breath, and milk and rapid loss of body condition.

Early detection can be helpful in preventing the heavy economic losses to farmers by ketosis and quick recovery of the animal.

## Authors’ Contributions

AK and SR proposed the study. GC, AK, and PK carried out the urine collection and diagnostic part. NS, TK, and AK critically observed the data and calculated mean and used SPSS statistical software. AK, GC, and S prepared the manuscript. Finalization of the manuscript was done by SR, NS, and VKJ. All authors read and approved the final manuscript.
